# Long-Term Outcomes in Crosslinking Therapy for Patients with Progressive Keratoconus

**DOI:** 10.3390/diagnostics15050626

**Published:** 2025-03-05

**Authors:** Tevfik Serhat Bahar, Vedat Şahin, Yusuf Ayaz, Mustafa Ünal

**Affiliations:** 1 Department of Ophthalmology, Antalya Korkuteli State Hospital, Antalya 07800, Turkey; 2 Department of Ophthalmology, Faculty of Medicine, Akdeniz University, Antalya 07070, Turkey

**Keywords:** keratoconus, corneal cross-linking, topography, epi-on, epi-off

## Abstract

**Background/Objectives:** To investigate the mid- and long-term postoperative follow-up results of corneal crosslinking (CXL) treatment (using epi-on and epi-off techniques) in progressive keratoconus. **Methods:** This was a retrospective single center study conducted between October 2013 and July 2020. Patients who received CXL treatment with a diagnosis of progressive keratoconus were included in the study. Preoperative and postoperative recorded examination findings of autorefractometry, visual acuity, endothelial cell counts by specular microscopy, and corneal topography were analyzed retrospectively. According to the latest measurements, the results were divided into two groups: measurements between 6 and 12 months and measurements between 1 and3 years. **Results:** A total of 290 operated eyes of 201 patients were included in the study. The mean age of the patients was 21.34 ± 5.77 years, and 119 (59.2%) were male. Epi-off CXL was performed on 269 (92.8%) eyes and epi-on procedure was performed on 21 (7.2%) eyes. CXL had no significant effect on visual acuity. Significant improvement was observed in topographic/keratometric features of the cornea both after 6–12 months and after 1–3 years. Significant decreases were observed in K1 (*p* < 0.001), K2 (*p* < 0.001), KM (*p* < 0.001) values compared to the initial level. **Conclusions:** CXL treatment is an important treatment method in the treatment of keratoconus, preserving visual functions, significantly reducing the severity of astigmatism, and stopping the progression of keratoconus. Additionally, although epi-off and epi-on CXL methods were compared in our study, the sample size was limited, and more comprehensive and long-term studies are needed.

## 1. Introduction

Keratoconus is a degenerative, non-inflammatory disease characterized by progressive corneal protrusion and thinning, causing vision impairment and corneal scarring [1,2]. It occurs in similar frequency among males and females, but demonstrates highest incidence during the second decade of life [1]. Prevalence ranges from 8.8 to 229 per 100,000 individuals, with relatively higher frequency in certain populations [3,4,5,6].

Multiple factors have considerable impact on the development of different types of keratoconus [7,8]; however, biomechanical and immunohistochemical studies have shown that matrix proteoglycans differ in normal cornea and keratoconic cornea [9,10]. The increase in proteolytic enzymes and the decrease in protease inhibitors may cause corneal pachymetric thinning and can alter stromal collagen [11,12]. In keratoconic corneas, vision loss may develop due to adverse effects on stromal fibrils and collagen lamellae [6].

Treatment alternatives of keratoconus include the use of glasses or contact lenses, corneal collagen crosslinking (CXL) treatment, and other surgical methods such as keratoplasty, intracorneal ring segments, photorefractive keratotomy, and radial keratotomy [1]. Each of these methods has different advantages, but their use is largely limited by their disadvantages. Glasses have limited benefit in the presence of uncorrectable irregular astigmatism, anisometropia, change of refractive error in a very short time, and high corneal toricity [13]. Contact lenses can be effective in some cases but they carry the potential for discomfort [13]. There is a high risk of astigmatism and graft rejection after some keratoplasty techniques [14,15]. Intracorneal ring segments are contraindicated in many cases and, even when they can be applied, these are single-use treatments which carry various risks [16].

Owing to the aforementioned limitations, corneal CXL has become the gold standard to prevent the progression of mild and moderate keratoconus by strengthening collagen matrix biomechanics [17]. This treatment is performed via the photopolymerization of stromal collagen fibers, which is based on the combined action of a photosensitizing substance (riboflavin or vitamin B2) and ultraviolet (UV) A light [1,18]. CXL was first used in the production and strengthening of prosthetic heart valves and dental filling material [19,20]. Later improvements and variations in the use of CXL led to its use in the treatment of keratoconus [1,21,22]. Although its efficacy in keratoconus has been demonstrated in many subsequent studies [23,24,25,26,27], the number of patients examined by these studies are often limited and patient follow-up is short [18,28,29,30,31,32].

Although used widely, B2 is a hydrophilic substance, and it is not very soluble in the corneal epithelium. For this reason, Spoerl et al. recommended debridement of the central corneal epithelium before CXL (epithelium off approach; epi-off) [22]. Later studies further suggested debridement of all layers of the corneal epithelium in order to allow for sufficient stromal B2 concentration after administration of 0.1% B2 [33,34]. As a result of these studies, the epi-off method has been accepted as the gold standard treatment method. The most common complications after epi-off corneal CXL are corneal haze, scar formation, infectious and non-infectious keratitis, ulcers, and endothelial damage [17]. These are considerable risks and related concerns led to the development of non-debridement methods (epi-on), which have been demonstrated to reduce postoperative pain, risk of infection, scar formation in the cornea, and endothelial damage [35,36]. However, studies comparing the outcomes of epi-on and epi-off methods report contradictory results [37,38,39,40,41], indicating the need for further studies that would enable better patient management.

In this study, we aimed to present our mid- and long-term results in patients who underwent corneal CXL due to progressive keratoconus, and to compare the success of epi-on and epi-off methods in preventing keratoconus progression and correcting vision problems.

However, the difference between our study and other studies is that it was a long-term study with a sufficient number of patients.

## 2. Material and Methods

### 2.1. Study Design, Setting, and Ethics

This retrospective study was carried out in the Ophthalmology Department of Akdeniz University (Antalya, Turkey) Hospital between October 2013 and July 2020. Approval for the study was received from the local ethics committee (date: 24 August 2022, no: KAEK-513). All procedures performed were in accordance with the 1964 Helsinki declaration and its later amendments.

### 2.2. Inclusion and Exclusion Criteria

The inclusion criteria for the study were: (i) Having progressive keratoconus disease [defined as maximum keratometry (Kmax) increase of 1 Diopter (D) or more, corneal astigmatism increase of 1 D or more, and increase in manifest refraction spherical equivalent (MRSE) of 0.50 D or more—verified by consecutive examination with a 3-month interval [42]; (ii) being under 20 years of age at diagnosis; (iii) having a transparent cornea; (iv) detection of a corneal thickness of ≥400 nm at the thinnest point; (v) not having any ocular disease other than keratoconus.

Exclusion criteria were: (i) History of previous refractive surgery; (ii) history of herpetic keratitis or presence of corneal scarring; (iii) pregnancy or breastfeeding (at baseline or follow-up); (iv) corneal infection or accompanying autoimmune disease; (v) presence of severe dry eye or any connective tissue disorders; (vi) missing data; (vii) missing follow-up data or insufficient follow-up.

### 2.3. Data Collection

Preoperative and postoperative examinations were recorded retrospectively, including autorefractometry, visual acuity, endothelial cell counts by specular microscopy, and corneal topography.

Uncorrected distance visual acuity (UDVA) and corrected distance visual acuity (CDVA) measurements were made using the Snellen chart from a distance of 6 m under the same lighting conditions. Visual acuity was recorded and analyzed as logMAR value [43]. 

Spherical power, cylindrical power, and apparent refractive spherical equivalent (MRSE) were measured with a Topcon KR 800 autorefractometer (Topcon, Japan).

Central corneal endothelial cell density was measured with a KONAN Cellchek 9900 Specular Microscope (Konan Medical USA Inc., Irvine, CA, USA). Three endothelial images of each eye were selected for analysis and averaged [44].

Corneal topography measurements were obtained using a rotating Scheimpflug camera (Pentacam; Oculus GmBH, Wetzlar, Germany). It was used to record flat (K1), steep (K2), and mean (Km) simulated keratometric values, central corneal thickness, corneal volume, maximum height values in anterior and posterior elevation maps, anterior chamber volume, anterior chamber depth, anterior chamber angle, and cylindrical dioptric power values.

Each of these measurements were performed at pre-scheduled visits for 3 years throughout the follow-up examinations. Based on the latest measurements obtained from patients, the results were divided into two groups: measurements between 6–12 months and measurements between 1–3 years.

### 2.4. Surgical Procedures

#### 2.4.1. Epi-Off CXL

After reducing pupil size with pilocarpine and applying topical anesthesia (proparacaine hydrochloride 0.5%), the corneal epithelium (8–10 mm diameter) was gently removed. A 0.1% topical B2 solution was applied to the stroma every 2–3 min for 30 min. Following stromal saturation, UVA irradiation (3 mW/cm^2^) began, lasting 30 min with 5 min intervals of topical B2. Post-procedure, antibiotic drops were administered, a bandage contact lens was placed, and the eye was covered. Patients were scheduled for a follow-up the next day.

#### 2.4.2. Epi-On CXL

Patients who wore contact lenses were advised to discontinue their use for two weeks prior to the procedure. Before the procedure, the eye was numbed using proparacaine hydrochloride 0.5%. A UVA device calibrated at 3 mw/cm^2^ was employed for 30 min without the removal of the corneal epithelium. Throughout the UVA treatment, topical B2 drops were consistently administered at 5 min intervals. Upon completion of the procedure, topical antibiotic drops were administered, and a bandage contact lens was placed.

#### 2.4.3. Postoperative Management

After the procedure, patients received topical moxifloxacin, with one drop every 8 h for the initial 5 days, followed by one drop every 5 h for the subsequent 5 days. In the three months following the operation, patients used non-preservative artificial tears, one drop, 4–6 times a day. Dexamethasone sodium phosphate drops were introduced at 4 times a day, beginning on the 4th day post-surgery and continued until the 10th day. Following the discontinuation of dexamethasone sodium phosphate drops on the 10th day, loteprednol drops were applied at a frequency of 4 times a day for 20 days. The bandage contact lens was removed once the epithelium had fully healed. Patients underwent follow-up appointments on the 1st day, 7th day, 1st month, 3rd month, 6th month, and subsequently at 6-month intervals.

### 2.5. Statistical Analysis

Descriptive statistics were performed/calculated to summarize the data. Categorical variables were expressed as frequencies (percentage), normally distributed continuous variables were summarized with means and standard deviation, and non-normally distributed continuous variables were reported with median and interquartile range (IQR). We assessed continuous data for its normality of distribution by using the Shapiro–Wilk test. Preoperative and postoperative measurements were compared using repeated measures analysis of variance (ANOVA) for normally distributed variables, while the Friedman test was used for non-normally distributed variables. The epi-on and epi-off groups were compared using independent samples t-tests for normally distributed variables and the Mann–Whitney U tests for non-normally distributed ones—with Bonferroni correction applied for pairwise comparisons. All statistical analyses were carried out using IBM SPSS Statistics Version 23.0, with statistical significance set at *p* values below 0.05.

## 3. Results

A total of 290 operated eyes of 201 patients were included in the study. The mean age of the patients was 21.34 ± 5.77 years, and 119 (59.2%) were male. Epi-off CXL was performed on 269 (92.8%) eyes and epi-on procedure was performed on 21 (7.2%) eyes (Table 1).

### 3.1. Changes in Visual Acuity and Refraction

For all eyes, no significant differences were observed between preoperative UDVA and CDVA and those at 6–12 months (*p* = 0.518) and 1–3 years (*p* = 0.185) postoperatively. Median spherical power and MRSE measured at 6–12 months postoperatively and 1–3 years postoperatively were significantly lower than preoperative values (*p* < 0.001 for both). There was a significant decrease in median cylindrical power measured at postoperative 1–3 years compared to those at the preoperative period (*p* = 0.015) (Table 2).

### 3.2. Changes in Endothelial Cell Density

Median endothelial cell density measured at 6–12 months postoperatively and 1–3 years postoperatively was statistically lower compared to preoperative values, and those measured at 1–3 years postoperatively compared to those measured at 6–12 months (*p* < 0.001 for both) (Table 3).

### 3.3. Changes in Topographic Measurements

**Figure 1:**
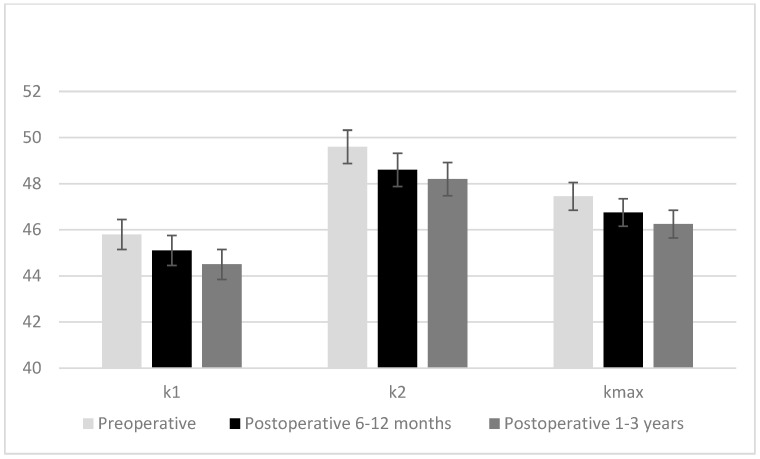
Changes in K1, K2, and Km values of the cases preoperative, postoperative 6–12 months, and postoperative 1–3 years.

While the mean corneal volume, anterior chamber volume, anterior chamber depth, and median maximum anterior elevation and maximum posterior elevation values measured at postoperative 6–12 months and postoperative 1–3 years were significantly lower than preoperative values, the mean anterior chamber angle was significantly higher (*p* < 0.001 for all). While the mean corneal volume, anterior chamber volume, anterior chamber depth, and median maximum anterior elevation values measured at the postoperative 1–3 years were significantly lower than the values in the postoperative 6–12 months, the mean anterior chamber angle was significantly higher (*p* < 0.001 for all). Median K1, K2, and Km values measured at 6–12 months postoperatively and 1–3 years postoperatively were lower than preoperative values, and the K1, K2, and Km values measured 1–3 years postoperatively were lower than the values measured at 6–12 months postoperatively (*p* < 0.001 for all) (Figure 1). The median central corneal thickness in the postoperative 6–12 months and postoperative 1–3 years was significantly lower compared to the preoperative one, and the median central corneal thickness in the postoperative 1–3 years was significantly lower compared to the postoperative 6–12 months (*p* < 0.001 for both) (Table 4).

### 3.4. Epi-Off Versus Epi-On

Almost all of the above-mentioned significant changes were also observed in the epi-off group. The only exception was that in the epi-off group, there was no significant difference between mean anterior chamber depth measured at 1–3 years postoperatively and those measured at 6–12 months. In the epi-on group, the median cylindrical power measured 6–12 months postoperatively was significantly higher than preoperative data (*p* = 0.004). Median endothelial cell density and mean central corneal thickness measured at 6–12 months and 1–3 years postoperatively were significantly lower than those measured preoperatively (*p* < 0.001 for both). The mean anterior chamber volume measured 1–3 years postoperatively was significantly lower than that measured preoperatively (*p* = 0.009), while the mean anterior chamber angle was significantly higher (*p* = 0.003). The median endothelial cell density (*p* = 0.003 and *p* = 0.002, respectively), median K1 (*p* = 0.012 and *p* = 0.002, respectively), and median Km (*p* = 0.040 and *p* = 0.007, respectively) measured at 6–12 months and 1–3 years postoperatively were significantly lower in the epi-off group than in the epi-on group. The median K2 of the epi-off group measured at 1–3 years postoperatively was significantly lower than that of the epi-on group (*p* = 0.043).

Corneal clouding developed in 13 eyes (4.8%) who underwent epi-off CXL, and epithelial toxicity and corneal scarring were not observed in the operated patients. Keratoconus progression was observed in 11 (4.1%) patients who underwent epi-off CXL. None of the patients who underwent epi-On CXL developed corneal turbidity, epithelial toxicity, and corneal scarring. Keratoconus progression was observed in 1 (4.7%) patient who underwent epi-on CXL.

## 4. Discussion

In the early 2000s, corneal CXL with riboflavin was introduced to increase corneal resistance in patients diagnosed with progressive keratoconus [23,45]. In theory, CXL treatment should enhance the biomechanical properties of the corneal stroma, thereby preventing the progression of keratoconus and ectasia. Subsequently, numerous studies have provided support for this theoretical framework [23,24,25,26]. In this study, we aimed to present our clinical results, including changes in visual acuity, refraction, endothelial cell density, and topographic features within 3 years after CXL, and to present the differences in the changes in these properties between epi-on and epi-off techniques. Our main findings showed that CXL had no significant effect on visual acuity in eyes with progressive keratoconus, but significantly improved refractive parameters, topographic features of the cornea, and endothelial cell count at both intermediate and long-term follow-up. Nonetheless, despite largely similar results, compared to the epi-on approach, the epi-off technique achieved significantly better outcomes in measurements other than visual acuity.

In this study, no significant difference was observed between UDVA and CDVA values before and after CXL (both measurements between 6 and 12 months and measurements between 1 and 3 years). Results of a retrospective study showed that 12 months after magnetic CXL treatment for progressive keratoconus, CDVA improved in at least one line in 54% of the eyes and remained stable in 28% [35]. In a retrospective uncontrolled double-center study, it was shown that 6 months after corneal CXL treatment, CDVA stabilized in 48.1% of the eyes, improved in 32.7% of the eyes, and decreased in 16.3% of the eyes. After 12 months, CDVA stabilized in 47.6% of the eyes, improved in 40.0% of the eyes, and decreased in 12% of the eyes [24]. In the study of Buzzonetti et al., it was reported that CDVA significantly improved with transepithelial CXL at the 18-month follow-up, whereas K readings and higher order aberrations significantly worsened [31]. Greval et al. reported no statistically significant difference in visual acuity in the 1-year follow-up of patients to whom CXL was performed using the epi-off method [46]. It was shown in a randomized, prospective study that in treated eyes at 18 months, CDVA improved after CXL treatment [28]. Hersh and colleagues investigated the 1-year results of CXL in patients with keratoconus or corneal ectasia. The results of the study showed that CXL was effective in improving UDVA and CDVA. CDVA worsened between baseline and 1 month, but then improved and then stabilized between 1, 3, and 6 months [29]. These and many other studies [18,32,47], as well as the current study, reveal that visual acuity either does not change or improves after CXL. This improvement seems to be more evident in the medium and long term compared to the short term.

In our study, a significant decrease was observed in MRSE values and spherical power measured at postoperative 6–12 months and 1–3 years compared to preoperative values, while there was no significant difference between the values at postoperative 6–12 months and 1–3 years. Cylindrical power also decreased in 1–3 years post-operatively compared to baseline. In a prospective case series involving patients with progressive keratoconus treated with CXL, spherical equivalent improved significantly after 1 and 2 years compared to baseline [32]. Another study showed that spherical equivalent increased in the first postoperative month, then showed hyperopic shifts after 1, 2, 3, and 4 years. The reduction in MRSE values became statistically significant after 12 months [18]. CXL appears to provide significant improvements in refraction impairment, especially regarding spherical equivalents.

The current study showed that endothelial cell density in the postoperative 6–12 month and 1–3 year periods were significantly lower than preoperative values; moreover, data from the 1–3 year period displayed significantly lower values compared to the 6–12 month period, indicating that endothelial cell density decreases over time. Ultraviolet (UV) rays used in crosslinking treatment can damage corneal endothelial cells. UV rays can damage the DNA of cells and cause cell death. Riboflavin used in the treatment interacts with UV rays to form free radicals. These free radicals can damage endothelial cells and cause cell death. At the same time, the heat generated during crosslinking can damage endothelial cells. Heat can disrupt cell membranes and protein structures, leading to cell death. Mechanical pressure and manipulations applied to the cornea during crosslinking can damage endothelial cells. All these reasons are thought to be among the reasons for the decrease in endothelial cell density. Caporossi and colleagues showed that treatment resulted in a non-significant increase in the loss of endothelial cells compared to the physiological baseline [18]. It also appears that this deleterious effect, if any, begins after some delay, as demonstrated by the study by Asri et al., who found similar endothelial cell counts at postoperative 6 and 12 months compared to preoperative values [24]. Furthermore, Goldich et al. reported that preoperative values were not significantly different from endothelial cell density values measured at the 6th, 12th, and 24th months postoperatively [48]. Unlike the mentioned studies, the current study showed that CXL treatment (both epi-on and epi-off) resulted in a permanent decrease in the number of endothelial cells at the end of 3-year follow-up. This effect has been associated with UVA dosage and cytotoxic effects. However, it should be emphasized that the endothelium is generally unaffected, except in cases of hydrops.

In the current study, according to the topographic evaluation, a number of parameters (corneal volume, anterior chamber volume and depth, maximum anterior elevation, K1, K2, and Km values, and central corneal thickness) in the postoperative 6–12 months and postoperative 1–3 years were significantly lower than the values in the preoperative period, and this time-bound decrease continued to be significant in the follow-up period (postoperative 1–3 years data were lower compared to 6–12 months). Anterior chamber angle values were lowest at baseline, followed by postoperative 6–12 months, and highest in postoperative 1–3 years. Additionally, maximum posterior elevation values were lower in both postoperative measurements compared to preoperative data. Postoperative cylindrical dioptric power was similar in postoperative and preoperative assessments. Our results are largely consistent with the literature. Many studies have reported that central corneal thickness [25,26,27], flat, steep, and mean curvature power of the cornea [26,49,50], anterior chamber measurements [26,51,52], corneal volume [51,53] and maximum anterior and/or posterior elevation [54,55,56] either significantly improved or stopped progressing in the short, medium, or long term after CXL.

The classical CXL protocol was an epi-off procedure. In this procedure, the central corneal epithelium was elevated by approximately 8 mm and riboflavin solution was applied to the exposed corneal stroma [39]. The CXL epi-off was eventually modified in favor of a technique that does not involve epithelial debridement, the so-called “epi-on” or transepithelial CXL method [57]. Although this new approach was developed to reduce the postoperative side effects of traditional epi-off CXL, the risk of failure is a crucial limitation of this method [1,39]. In our study, epi-on CXL was applied to 21 patients and epi-off CXL was applied to 269 patients. While all refraction parameters improved over time in patients treated with epi-off, epi-on CXL only caused a significant increase in cylindrical power in 6–12 months. Both techniques provided a significant reduction in endothelial cell density at both postoperative periods. The reduction achieved with the epi-off technique increased significantly over time. Most of the topographic parameters improved over time with the epi-off technique, but the improvements observed for the epi-on technique were limited to central corneal thickness, anterior chamber angle, and anterior chamber volume. A systematic review and meta-analysis showed no statistical significance in visual acuity in epi-on vs. epi-off CXL at the 1-year follow-up. It was shown that the reduction in Km was greater with epi-off CXL when compared to the epi-on CXL [58]. In a study of pediatric population, patients undergoing epi-on iontophoresis CXL showed no improvement in CDVA from baseline at 3 years follow-up, while those receiving epi-off CXL showed significant improvements in CDVA [37]. In a 5-year pediatric prospective study, the epi-off group showed a significant cylindrical decrease at 12 and 60 months [38]. Cifariello et al. reported significant improvements for vision, corneal thickness, and nerve features in both the epi-off and epi-on groups. Notably, K flat, K steep, and CDVA values 2 years after CXL were significantly lower in the epi-off group [39].

Various studies have reported that both epi-on and epi-off treatments similarly halt the progression of keratoconus [41,59]. However, various studies have shown that patients treated with epi-on CXL experience greater keratoconus progression [60] and worse stabilization rates [61,62] compared to epi-off CXL. Despite the fact that some studies have favored epi-off based on keratoconus outcomes, others demonstrated that visual improvement is faster with epi-on and that pain and infection rates associated with epithelial defects are rarer [41,59,61,62]. It is possible that the results favoring epi-off were associated with the fact that experience with epi-off was much more extensive until the widespread use of epi-on. Nonetheless, the results of our study also suggest that epi-off CXL is better in preventing keratoconus progression, with the added benefit of dramatic improvement in vision-related outcomes. But importantly, in this study, only a fraction of the patients underwent epi-on CXL, which could have affected the outcomes and their statistical comparison results.

### Limitations

Some limitations of the study should be taken into consideration when interpreting the results. The fact that it is a single-center study with few epi-on procedures limits its external validity. The initial retrospective design restricted the addition and exploration of new data during follow-up. The epi-off and epi-on techniques could not be compared in terms of adverse events due to unstandardized records for complications or adverse outcomes. However, the patient count is greater than many similar studies in the field, but the aforementioned bias in distribution could have impacted analyses. Since the study did not include patient groups receiving other treatments, CXL could not be compared with other methods.

## 5. Conclusions

Our data showed that no significant change in visual acuity was seen after CXL in patients with keratoconus, despite some significant improvements in refraction measurements. Additional advantages were shown in topographic/keratometric parameters and endothelial cell density, all of which demonstrated an increasing trend from 6–12 months to 3 years. The improvements achieved via epi-off CXL in terms of endothelial cell count and keratometric measurements were significantly more pronounced compared to epi-on CXL. However, this observation should be supported by studies that included a greater number of epi-on patients. CXL treatment is an important treatment for keratoconus that preserves visual functions, significantly reduces the severity of astigmatism caused by keratoconus, and stops the progression of keratoconus. More comprehensive and prospective studies with long-term follow-ups are needed to compare the efficacy and outcomes of epi-off and epi-on CXL.

## Figures and Tables

**Table 1:** Summary of age, sex, and operation (290 eyes of the 201 patients).

Age	21.34 ± 5.77
Sex	
Male	119 (59.2%)
Female	82 (40.8%)
Operation	
Epi-Off	269 (92.8%)
Epi-On	21 (7.2%)

Descriptive statistics were represented with frequency (percentage) for categorical variables and mean ± standard deviation for normally distributed continuous variables.

**Table 2:** Comparison of visual acuity and refractive changes in all patients in the epi-off and epi-on groups before surgery, 6–12 months after surgery, and 1–3 years after surgery.

		Operation		
	All Eyes (*n* = 290)	Epi-Off (*n* = 269)	Epi-On (*n* = 21)	*p* (Between Groups)
Uncorrected distance visual acuity (logMAR)				
Preoperative	0.5 (0.3–1.0)	0.5 (0.3–1.0)	0.5 (0.4–1.0)	0.713
Postoperative 6–12 months	0.5 (0.3–0.7)	0.5 (0.3–0.7)	0.5 (0.4–0.7)	0.654
Postoperative 1–3 years	0.5 (0.3–0.7)	0.5 (0.3–0.7)	0.5 (0.2–0.7)	0.643
*p* (within groups)	0.518	0.572	0.535	
Corrected distance visual acuity (logMAR)				
Preoperative	0.3 (0.2–0.5)	0.3 (0.2–0.5)	0.3 (0.2–0.4)	0.418
Postoperative 6–12 months	0.3 (0.2–0.5)	0.3 (0.2–0.5)	0.4 (0.2–0.5)	0.842
Postoperative 1–3 years	0.3 (0.2–0.5)	0.3 (0.2–0.5)	0.3 (0.2–0.4)	0.506
*p* (within groups)	0.185	0.246	0.683	
Spherical power (D)				
Preoperative	−1.5 (−3.75–0.5)	−1.5 (−3.5–−0.5)	−2.0 (−4.0–−0.75)	0.570
Postoperative 6–12 months	−1.25 (−3.5–0.0) *	−1.25 (−3.5–0.0) *	−2.0 (−3.25–−0.5)	0.635
Postoperative 1–3 years	−1.25 (−3.0–0.0) *	−1.25 (−3.0–0.0) *	−1.75 (−4.0–−0.75)	0.228
*p* (within groups)	<0.001	<0.001	0.555	
Cylindrical power (D)				
Preoperative	−3.75 (−6.0–2.25)	−3.87 (−6.0–−2.5)	−3.5 (−5.0–−1.25)	0.104
Postoperative 6–12 months	−3.75 (−5.75–2.5)	−3.75 (−5.87–−2.5)	−4.0 (−5.75–−2.75) *	0.711
Postoperative 1–3 years	−3.5 (−5.5–2.25) *	−3.5 (−5.5–−2.25) *	−3.25 (−6.25–−2.25)	0.938
*p* (within groups)	0.015	0.030	0.004	
Manifest refraction spherical equivalent (D)				
Preoperative	−3.75 (−5.87–−2.00)	−3.75(−5.94–−2.12)	−3.75 (−5.0–−1.37)	0.606
Postoperative 6–12 months	−3.75 (−5.75–1.75) *	−3.5 (−5.75–−1.75) *	−4.5 (−5.75–−1.88)	0.613
Postoperative 1–3 years	−3.12 (−5.37–1.62) *	−3.0 (−5.25–−1.62) *	−4.25 (−6.37–−1.87)	0.227
*p* (within groups)	<0.001	<0.001	0.424	
Cylindrical dioptric power (D)				
Preoperative	3.7 (2.5–5.2)	3.7 (2.5–5.2)	3.5 (2.7–4.5)	0.756
Postoperative 6–12 months	3.8 (2.5–5.2)	3.8 (2.5–5.2)	3.2 (2.5–4.7)	0.338
Postoperative 1–3 years	3.8 (2.5–5.2)	3.8 (2.5–5.3)	3.2 (2.3–4.8)	0.395
*p* (within groups)	0.905	0.890	0.282	

Descriptive statistics were represented with mean ± standard deviation for normally distributed continuous variables and median (25th percentile–75th percentile) for non-normally distributed continuous variables. *: Significantly different from “Preoperative”. The Shapiro–Wilk test was used to evaluate continuous data in terms of normal distribution. Abbreviation: D—Diopter, LogMAR—Logarithm of the Minimum Angle of Resolution.

**Table 3:** Comparison of endothelial cell density changes in all patients in the epi-off and epi-on groups before surgery, 6–12 months after surgery, and 1–3 years after surgery.

	Operation			
All Eyes (*n* = 290)	Epi-Off (*n* = 269)	Epi-On (*n* = 21)	*p* (Between Groups)	
Endothelial cell density (cells/mm^2^)				
Preoperative	2841 (2674–3012)	2841 (2674–3003)	2915 (2688–3021)	0.547
Postoperative 6–12 months	2538 (2315–2710) *	2497 (2312–2695) *	2710 (2604–2857) *	0.003
Postoperative 1–3 years	2432.5 (2234–2639) *^#^	2397 (2232–2596) *^#^	2639 (2555–2717) *	0.002
*p* (within groups)	<0.001	<0.001	<0.001	

Descriptive statistics were represented with mean ± standard deviation for normally distributed continuous variables and median (25th percentile–75th percentile) for non-normally distributed continuous variables. *: Significantly different from “Preoperative”, #: Significantly different from “Postoperative 6–12 months”. Abbreviation: D—Diopter, LogMAR—Logarithm of the Minimum Angle of Resolution. The Shapiro–Wilk test was used to evaluate continuous data in terms of normal distribution.

**Table 4:** Comparison of changes in topographic measurements in all patients in the epi-off and epi-on groups before surgery, 6–12 months after surgery, and 1–3 years after surgery.

	Operation			
All Eyes (*n* = 290)	Epi-Off (*n* = 269)	Epi-On (*n* = 21)	*p* (Between Groups)	
Corneal volume (mm^3^)				
Preoperative	56.91 ± 3.29	56.96 ± 3.35	56.27 ± 2.28	0.351
Postoperative 6–12 months	56.28 ± 3.37 *	56.30 ± 3.43 *	55.99 ± 2.49	0.683
Postoperative 1–3 years	55.98 ± 3.39 *^#^	56.01 ± 3.45 *^#^	55.54 ± 2.47	0.428
*p* (within groups)	<0.001	<0.001	0.071	
Anterior chamber volume (mm^3^)				
Preoperative	201.99 ± 32.83	202.46 ± 32.69	196.00 ± 34.87	0.386
Postoperative 6–12 months	199.47 ± 32.49 *	199.85 ± 32.30 *	194.67 ± 35.23	0.482
Postoperative 1–3 years	197.14 ± 32.66 *^#^	197.62 ± 32.48 *^#^	190.90 ± 35.14 *	0.365
*p* (within groups)	<0.001	<0.001	0.009	
Anterior chamber depth (mm)				
Preoperative	3.39 ± 0.28	3.38 ± 0.28	3.40 ± 0.28	0.838
Postoperative 6–12 months	3.36 ± 0.29 *	3.36 ± 0.29 *	3.41 ± 0.26	0.477
Postoperative 1–3 years	3.35 ± 0.30 *^#^	3.35 ± 0.30 *	3.38 ± 0.28	0.640
*p* (within groups)	<0.001	<0.001	0.155	
Anterior chamber angle (degree)				
Preoperative	38.21 ± 6.19	38.21 ± 6.24	38.15 ± 5.55	0.963
Postoperative 6–12 months	40.36 ± 5.78 *	40.46 ± 5.73 *	39.18 ± 6.43	0.331
Postoperative 1–3 years	41.03 ± 6.02 *^#^	41.11 ± 5.98 *^#^	39.95 ± 6.56 *	0.396
*p* (within groups)	<0.001	<0.001	0.003	
Maximum anterior elevation (µm)				
Preoperative	13 (8–20)	13 (8–19)	14 (6–20)	0.688
Postoperative 6–12 months	12 (6–17) *	12 (6–17) *	13 (4–17)	0.840
Postoperative 1–3 years	9.5 (4–15) *^#^	9 (4–15) *^#^	11 (5–16)	0.479
*p* (within groups)	<0.001	<0.001	0.095	
Maximum posterior elevation (µm)				
Preoperative	30 (18–46)	31 (19–46)	28 (13–42)	0.585
Postoperative 6–12 months	29 (18–44) *	29 (18–44) *	28 (14–40)	0.696
Postoperative 1–3 years	29 (19–43) *	29 (19–43) *	29 (14–41)	0.789
*p* (within groups)	0.001	<0.001	0.695	
Flat curvature power of the cornea (D)				
Preoperative	45.8 (44.1–47.8)	45.7 (44.1–47.7)	46.1 (45.3–48.0)	0.098
Postoperative 6–12 months	45.1 (43.2–47.5) *	45.0 (43.0–47.2) *	46.5 (44.7–49.5)	0.012
Postoperative 1–3 years	44.5 (42.5–46.6) *^#^	44.2 (42.2–46.3) *^#^	46.6 (44.7–48.8)	0.002
*p* (within groups)	<0.001	<0.001	0.243	
Steep curvature power of the cornea (D)				
Preoperative	49.6 (47.5–52.5)	49.6 (47.5–52.4)	49.3 (48.4–53.8)	0.293
Postoperative 6–12 months	48.6 (46.6–52.2) *	48.5 (46.6–51.8) *	48.9 (48.0–53.5)	0.142
Postoperative 1–3 years	48.2 (45.8–50.9) *^#^	48.1 (45.7–50.8) *^#^	49.7 (48.0–53.4)	0.043
*p* (within groups)	<0.001	<0.001	0.091	
Mean curvature power of the cornea (D)				
Preoperative	47.45 (46.0–50.1)	47.4 (45.9–50.0)	48.1 (46.7–50.8)	0.185
Postoperative 6–12 months	46.75 (44.9–49.6) *	46.7 (44.9–49.4) *	48.0 (46.3–50.9)	0.040
Postoperative 1–3 years	46.25 (44.2–48.5) *^#^	46.1 (44.1–48.4) *^#^	47.7 (46.3–50.7)	0.007
*p* (within groups)	<0.001	<0.001	0.176	
Central corneal thickness (µm)				
Preoperative	474.85 ± 32.68	475.65 ± 32.85	464.57 ± 29.14	0.135
Postoperative 6–12 months	450.79 ± 40.65 *	450.83 ± 41.56 *	450.19 ± 26.87 *	0.921
Postoperative 1–3 years	445.56 ± 42.86 *^#^	445.39 ± 43.95 *^#^	447.76 ± 25.55 *	0.704
*p* (within groups)	<0.001	<0.001	<0.001	

Descriptive statistics were represented with mean ± standard deviation for normally distributed continuous variables and median (25th percentile–75th percentile) for non-normally distributed continuous variables. *: Significantly different from “Preoperative”, #: Significantly different from “Postoperative 6–12 months”. Abbreviation: D—Diopter, LogMAR—Logarithm of the Minimum Angle of Resolution. The Shapiro–Wilk test was used to evaluate continuous data in terms of normal distribution.

## Data Availability

The data underlying this article will be shared upon reasonable request to the corresponding author.
